# Bottleneck in secretion of α-amylase in *Bacillus subtilis*

**DOI:** 10.1186/s12934-017-0738-1

**Published:** 2017-07-19

**Authors:** Shaomin Yan, Guang Wu

**Affiliations:** National Engineering Research Center for Non-food Biorefinery, Guangxi Academy of Sciences, 98 Daling Road, Nanning, 530007 Guangxi China

**Keywords:** α-Amylase, *B. subtilis*, Chaperone, Production, Secretion

## Abstract

Amylase plays an important role in biotechnology industries, and Gram-positive bacterium *Bacillus subtilis* is a major host to produce heterogeneous α-amylases. However, the secretion stress limits the high yield of α-amylase in *B. subtilis* although huge efforts have been made to address this secretion bottleneck. In this question-oriented review, every effort is made to answer the following questions, which look simple but are long-standing, through reviewing of literature: (1) Does α-amylase need a specific and dedicated chaperone? (2) What signal sequence does CsaA recognize? (3) Does CsaA require ATP for its operation? (4) Does an unfolded α-amylase is less soluble than a folded one? (5) Does α-amylase aggregate before transporting through Sec secretion system? (6) Is α-amylase sufficient stable to prevent itself from misfolding? (7) Does α-amylase need more disulfide bonds to be stabilized? (8) Which secretion system does PrsA pass through? (9) Is PrsA ATP-dependent? (10) Is PrsA reused after folding of α-amylase? (11) What is the fate of PrsA? (12) Is trigger factor (TF) ATP-dependent? The literature review suggests that not only the most of those questions are still open to answers but also it is necessary to calculate ATP budget in order to better understand how *B. subtilis* uses its energy for production and secretion.

## Background

Amylase, especially α-amylase, is an important biological product with its wide applications in clinical practice [1, 2] and in industry [3, 4]. For this reason, bacteria are considered as an important cell factory to produce α-amylase [5], especially a Gram-positive bacterium, *Bacillus subtilis*, because of its high yield of designed proteins [6, 7], its free of endotoxins, and its simplicity in terms of a few hundreds of proteins making up a viable cell [8].

Although *B. subtilis* frequently serves as a model bacterium to enrich our knowledge on how different mechanisms work in bacteria [9], it does produce desired products economically and profitably, such as riboflavin [10–12], branched-chain amino acids [13], amylase [14], etc.

To increase the production of α-amylase in *B. subtilis*, heterologous α-amylases are introduced into *B. subtilis*, for instances, AmyL comes from *B. licheniformis* [15], AmyM and AmyS come from *Geobacillus stearothermophilus* [14], and AmyQ comes from *B. amyloliquefaciens* [16]. These introductions are necessary because, for example, *B. amyloliquefaciens* demonstrates a strong ability in secretion but a weak ability in production, whereas *B. subtilis* does oppositely, and *B. amyloliquefaciens* has a bigger growth rate with larger cell density than *B. licheniformis* [17]. But most importantly, *B. subtilis* is used because its genetics is well known [8] and there are many molecular tools to manipulate it [8, 18].

However, incompatibility between α-amylase production and secretion is observed, and becomes the bottleneck in industrial production of α-amylase, and is termed as the secretion stress [19]. Primarily the secretion stress goes through three steps, the overproduction and hyper-secretion of α-amylases upregulate the secretion stress-responsive CssR–CssS regulatory system, then the CssR–CssS regulatory system upregulates HtrA and HtrB proteases [14, 19], and the proteases in turn degrade the secreted α-amylases via the Sec secretion system. Their consequence is the loss of secreted α-amylases. Hence, this bottleneck is mainly related to three systems (i) Sec secretion system, (ii) CssR–CssS regulatory system, and (iii) HtrA and HtrB system.

So far, numerous investigations have been carried out to address this bottleneck from different angles not only because these three systems themselves are composed of many components but also because these components are involved in many processes. These studies are performed at signal peptide level [20–22], at chaperone level [23–25], at protease level [26, 27], at folding level [28, 29], at promoter level [30–33], at DNA level [34], at transcriptome level [35, 36], at secretion system level [37], at stress response level [38, 39], at fermentation level [14], etc.

Collectively, the bottleneck has been studied in great details although unsolved problems are still there. For example, it has been reported that overexpression of secretion systems is time-consuming and not efficient in *B. subtilis* for α-amylase production [15, 40]. Since the secretion stress looks like a chain reaction, that is, it first begins from Sec secretion system and ends up to HtrA and HtrB system. Sec secretion system draws a considerable attention [15] although the other secretion systems also appear on radar screen [37]. Probably, the most efficient and effective way to deal with the bottleneck should start with Sec secretion system since it performs well before the initiation of secretion stress. Moreover, α-amylase is a secretory protein whose secretion process should tightly relate to each step of secretion in *B. subtilis*.

At this point, questions come out and require answers and further studies in light of α-amylase secretion and production. In particular, the following long-standing questions have yet to be answered. (1) Does α-amylase need a specific and dedicated chaperone? (2) What signal sequence does CsaA recognize? (3) Does CsaA require ATP for its operation? (4) Does an unfolded α-amylase is less soluble than a folded one? (5) Does α-amylase aggregate before transporting through Sec secretion system? (6) Is α-amylase sufficient stable to prevent itself from misfolding? (7) Does α-amylase need more disulfide bonds to be stabilized? (8) Which secretion system does PrsA pass through? (9) Is PrsA ATP-dependent? (10) Is PrsA reused after folding of α-amylase? (11) What is the fate of PrsA? (12) Is trigger factor (TF) ATP-dependent? Thus, attempting to answer these questions through reviewing of literature is designed as the aim of this question-oriented review.

## Chaperone for α-amylase

Gram-positive bacteria have a single cytoplasmic membrane and it is generally considered that they have six secretion systems: (i) the Sec secretion system for unfolded proteins [29, 41, 42], (ii) the twin-arginine translocation (Tat) secretion system for folded proteins [43], (iii) the fimbrillin-protein exporter (FPE) for the proteins that form pilin-like structures [44], (iv) the flagella export apparatus (FEA) for the proteins that form the flagella hook, filament, and cap [45], (v) the timed holin pore for the fully folded endolysin that degrades the cell wall during the phage lytic cycle [46], and (vi) the ESAT-6/WXG100 secretion system (WSS) for the proteins that contain a WXG motif [47] including virulence factors in some pathogenic bacteria [48, 49].

Main commercial production of amylases is produced by *Bacillus* species including *B. acerans*, *B. acidocaldarius*, *B. amyloliquefaciens*, *B. licheniformis*, *B. stearothermophilus*, *B. subtilis* and *Bifidobacterium bifidum* [50]. For protein secretion, *B. subtilis* uses four pathways: Sec-SRP (signal recognition particle) pathway, Tat pathway, ABC transporters and pseudopilin export pathway [51], based on the studies on signal peptides [52]. The majority of produced α-amylases is secreted through Sec-SRP pathway, and the rest passes through Tat pathway and ABC (ATP-binding cassette) transporters.

The SRP, which is the ribosome-associated RNA–protein complex and defined as the only secretion-specific chaperone Ffh protein [53], binds to hydrophobic signal-anchor or signal sequence in nascent chains and targets them to the Sec translocon via interaction with its membrane receptor FtsY [54]. On the other hand, TF is also a cytosolic ribosome-bound protein [55].

In *B. subtilis*, CsaA could be a replacement of SecB to export preproteins, i.e. PrePhoB and ProOmpA [56, 57]. As a matter of fact, the existence of CsaA is not limited to *B. subtilis* because not only Gram-positive bacteria have SecB-like sequences [58] but also most archaea have homologues of CsaA [20, 59]. Thus, the question is whether CsaA can work as a specific and dedicated chaperone to carry α-amylase precursor to SecA in *B. subtilis*. The answer probably is not as shown by Müller et al. [56] that the export of most proteins remained unaffected when repressing the expression of the CsaA gene while only the export of two proteins, 19 and 36 kDa, was significantly reduced upon CsaA depletion, while α-amylase is about 69 kDa mass weight [29]. Furthermore, CsaA was found not to bind to the conserved SecB-binding domain in SecA [56]. So it would be hard to conclude that CsaA carries α-amylase precursors to SecA with the same efficiency. Interestingly, *Thermus thermophilus*, a Gram-negative eubacterium, has both SecB and CsaA [60], therefore it would be great enlightening to analyze how SecB and CsaA work in this species. Moreover, it is unclear whether CsaA recognizes signal peptides because CsaA is indifferent to YvaY precursor and mature YvaY [61].

SecA usually binds to preproteins with mildly hydrophobic signal sequences [62], so it was suggested that SecA could serve as chaperone in *B. subtilis* [63] because the level of cytosolic SecA in *B. subtilis* is relatively high [64]. Interestingly, it was found that sodium azide, an ATPase inhibitor on SecA, had no influence on α-amylase secretion in *B. amyloliquefaciens* as early as in 1975 [65]. This suggests that either α-amylase does not go through Sec secretion system in *B. amyloliquefaciens* because sodium azide inhibits the secretion of cytotoxin in *B. cereus* [66], or SecA does not serve as chaperone to bind α-amylase.

Preprotein translocation is a process requiring energy provided by the peripheral membrane-associated ATPase SecA, whose cycling repeats from ATP binding to hydrolysis to initiate the stage of further translocation process [20]. Thus, we may wonder whether the overproduction of α-amylase exhausts the ATP source in *B. subtilis* whose ATP-dependent SecA, DnaK and GroEL cannot work well for secretion. Also, it is not clear whether TF is dependent upon ATP? As the major function of TF, DnaK and GroEL is to fold proteins, it is likely that they do not work for Sec secretion system because Sec secretion system requires unfolded proteins with signal peptides [20, 67]. As a whole, it is unlikely that α-amylase has a specific and dedicated chaperone.

## Binding of signal peptide

In fact, α-amylase secretion is not limited to Sec secretion system. For example, *Corynebacterium glutamicum* R is also a Gram-positive bacterium producing heterologous proteins such as amylase [68], nuclease, protease [69], transglutaminase [70], epidermal growth factor [71], protein glutaminase [72], etc. A screening of secretory proteins on its genome shows that 108 of 405 candidate signal peptides are able to heterologously secrete an active-form α-amylase derived from *G. stearothermophilus*, including 98 Sec-type and 10 Tat-type peptides [73]. For these 98 Sec-type α-amylases in *C. glutamicum* R, their average length of signal peptides is 36.6 residues and longer than those of 166 Sec-type proteins in *B. subtilis* [20], which have 28 residues ranging from 19 to 44 residues. In *B. subtilis*, about 300 endogenous proteins including α-amylase are secreted through Sec system [74], and AmyE has 36 residues, MFAKRFKTSLLPLFAGFLLLFHLVLAGPAAASAETA [20]. Although the average length of signal peptides is about 35 residues for 10 Tat-type α-amylases in *C. glutamicum* R, AmyX in *B. subtilis* has 30 residues, MVSIRRSFEAYVDDMNIITVLIPAEQKEIM [20]. Therefore, these findings suggest that some α-amylases, like AmyX, are in the folded form before reaching secretion system because Sec secrets unfolded protein with signal peptide [20, 67] whereas Tat secrets folded protein with signal peptide [75, 76]. Of the identified extracellular proteins, about 80 proteins pass through the Sec secretion system [77], fewer proteins such as PhoD and YwbN pass through Tat system [78], and the rest proteins may pass through ABC transporters [52].

PrsA, which is a post-translocation chaperone [79, 80], helps the folding of proteins secreted from Sec secretion system [28, 29]. It is vital [23, 81] and is closely related to α-amylase production because its overproduction leads to the increase of α-amylase production in *B. stearothermophilus* [15, 23, 82, 83], but also leads to the increase in the production of proteases [23]. Curiously, the competition between α-amylase and PrsA has not been seen through secretion systems because PrsA as a lipoprotein should pass through either Sec or Tat secretion systems [20]. As a lipoprotein, PrsA needs signal peptide peptidase II to cut its signal peptides, and then moves across the membrane [20]. This is very intriguing because the signal peptides are generally applied to secretory proteins whereas chaperones are not subject to the classification of signal peptides.

The amount of proteins being synthesized in ribosomes would be proportional to occupied ribosomes, which could trigger the involvement of SRP and TF, however the results from the synthesis of α-amylase from *Pyrococcus furiosus*-F30 did not show many occupations [17]. Therefore it is not very clear what type of signal peptides for these two ribosome-bound chaperones.

Currently, the signal peptide sequences are classified into four types according to the sequence that is recognized by signal peptide peptidase [20], and consequently each type of signal peptides controls a preprotein to direct a specific secretion system. So at this point, we could not answer the question of what signal peptide sequence CsaA can recognize? Along this line, it is highly likely that α-amylase does not need a dedicated chaperone because our current knowledge so far suggests that each chaperone works for a specific secretion system whereas α-amylase can go through different secretion systems. Of course, the non-classical secretion systems constantly get attention, which do not need the signal peptides [37].

## Folding of α-amylase

It is no doubt that the rate of protein folding is a determinant factor for production of α-amylase [84], and so far the PrsA’s role in folding of α-amylase gets special attention. If we consider the possibility that a part of α-amylases has already folded before reaching Tat system, then folding activity should extend into cytosolic compartment, where even Sec secretion system is influenced by the capacity of preventing premature folding, aggregation or degradation of preproteins [85], i.e., PrsA works on the trans side of the cytoplasmic membrane but is anchored, whereas the rest of chaperones works on the cis side of the cytoplasmic membrane but are free.

The involvement of PrsA in α-amylase folding was known a longtime ago [23, 81, 86]. As PrsA is a 33-kDa lipoprotein enriched with lysine, and its N-terminal cysteine attaches cytoplasmic membrane with a thiol-linked diacylglycerol [87, 88], the questions raised here are whether PrsA is reused after finishing the folding of an α-amylase, and what is the fate of PrsA? Since the peptidoglycan has about 1300 disaccharides with about 20% of cross-linked peptide chains in *B. subtilis* [89], its permeability is limited to 25 kDa globular proteins [90]. Naturally, α-amylase is too large to go through the porous-structured cell wall with its 69 kDa mass weight [29]. The numbers of PrsA are 2 × 10^4^ per wild-type cell and 2 × 10^5^ per enhanced secretion cell [79]. Now, it is not aware whether PrsA needs ATP, therefore it is hard to know how folded proteins are released from PrsA because the binding of chaperone with the hydrophobic peptide segments of substrates is controlled by ATP binding and hydrolysis [91]. Likely, PrsA may have no need to reuse itself considering such a large number of PrsA in *B. subtilis*. As PrsA is anchored on cytoplasmic membrane, thus its distance to Sec secretion system should be significant otherwise there is no need to have so many PrsA.

Except for the role of PrsA in folding of α-amylase, another question raised here is whether there are factors with antifolding activity, which could lead to the accumulation of unfolded and misfolded α-amylase. This could be possible, because the cell wall with net negative charge influences protein folding [82], which is essentially not limited to α-amylase but also to other heterogeneous proteins like levansucrase [92, 93]. Yet, calcium also plays a role in folding of α-amylase [93]. *Bacillus* species can improve α-amylase yield by inactivating the *dlt* operon, which results in the absence of alanylation of teichoic acids [82]. Consequently, the negative charges increase at cell membrane leading to a higher affinity for cations, which favorite the stability of secreted proteins and catalyze their folding.

Nonetheless, either PrsA or calcium is relevant to trans side where the folding requires Skp, SurA, and PpiD, while TF, DnaK (HSP70) and GroEL (HSP60) to work in cis side. If we consider that most α-amylases should be folded on trans side, not much attention may need to pay the folding process in cis side, although TF is the first chaperone to interact with most newly synthesized proteins co-translationally [94], and can delay the co-translational folding of large proteins [95–97].

**Fig. 1:**
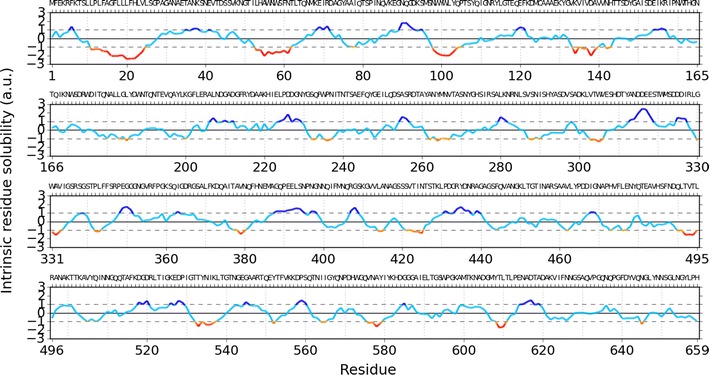
Intrinsic solubility score obtained from CamSol for α-amylase precursor, where a residue is less soluble when its score is less than unity (not *deep blue*)

DnaK also functions in stabilizing proteins for subsequent folding by GroEL. It is widely reported that a poorly folded protein forms inclusion bodies and is a very common phenomenon when protein folding systems are saturated. α-Amylases enable to aggregate before transporting through Sec secretion system. Also, unfolded α-amylase is less soluble than folded one because we find α-amylase precursor (accession number H9BPX5) poorly soluble when using CamSol [98] to compute its solubility (Fig. 1).

Although PrsA serves as a post-translocation chaperone to help the folding of secreted proteins and similar chaperones, peptidyl-prolyl cis/trans isomerase, exist in Gram-negative bacteria [99, 100], it seems that *B. subtilis* lacks the enzymes involving in disulfide bonds because PrsA is the only chaperone outside the cytoplasmic membrane [79]. In addition, their native disulfide bond-forming enzymes may have limited ability to form more disulfide bonds during the overproduction of heterologous proteins [101]. So a question raised here is whether α-amylase needs more disulfide bonds to be stabilized? Indeed, if we pick up randomly several α-amylases from *B. subtilis*, then we can find that not many cysteines in them. For example, each of A7DWA9, B8Y1H0, P00691, C0KWE6, G4F096 and G4NTU0 has one cysteine, each of G4PC62 and G4P8H4 has 2 cysteines, G4P133 has 3 cysteines, each of G4NTS1 and G4PA17 has 4 cysteines, G4P139 has 5 cysteines, and each of Q9R9H8, O06988 and G4P8I0 has 6 cysteines. As α-amylase lacks cysteins, which could form disulfide bonds to stabilize itself as other secreted proteins [102], so this could be a reason why there are misfolded α-amylase to be cleared by proteases. This is plausible because genes *trxA* and *trxB*, which prevent preproteins from oxidizing and to form disulfide bonds [103], are not active during overproduction in *B. subtilis*. The effectiveness of acylated homoserine lactone (AHL) with unsaturated C_18_ side chains was dependent on the number of double bonds in the acyl side chain [104]. In fact, not many secreted proteins in *B. subtilis* contain disulfide bonds [105]. Thus one may wonder whether the increase in thiol-disulfide oxidoreductase could be a way to stabilize α-amylase to reduce misfolded α-amylase. Indeed, it was showed that human interleukin-3 [106] and protease, both with a single disulfide bond, could be stable and efficiently secreted in *B. subtilis* [107]. On the contrary, it was showed in the past that human serum albumin and the human pancreatic α-amylase with several disulfide bonds were poorly secreted in *B. subtilis* [105, 108]. Clearly, more studies are needed in this regard.

## Conclusions

In this question-oriented review, we attempted to find the answers to 12 questions that we conceived during our study through reviewing of literature. The answers to these questions are not only important to biotechnology industry but also meaningful to theoretical studies in microbiology. At this point, we can briefly summarize our answers as follows:

1. Does α-amylase need a specific and dedicated chaperone? No.
2. What signal sequence does CsaA recognize? Unknown.
3. Does CsaA require ATP for its operation? Unknown.
4. Does an unfolded α-amylase is less soluble than a folded one? Yes.
5. Does α-amylase aggregate before transporting through Sec secretion system? Yes.
6. Is α-amylase sufficient stable to prevent itself from misfolding? No.
7. Does α-amylase need more disulfide bonds to be stabilized? Yes.
8. Which secretion system does PrsA pass through? Unknown.
9. Is PrsA ATP-dependent? Unknown.
10. Is PrsA reused after folding of α-amylase? Unknown.
11. What is the fate of PrsA? Unknown.
12. Is TF ATP-dependent? Unknown.

So an important implication is that can we develop models to better understand the expenditure of ATP in *B. subtilis* and what is the best 3D structure for α-amylase in order that it is stable and can be secreted smoothly without aggregation?

Hopefully our review can provide some clues to solve these pressing problems and advance our knowledge in this field. Indeed, many long-standing questions are eagerly waiting for their answers and inconsistent results remain to be addressed, and they are not obstacles for the development, but inspire the new endeavors. No doubt, the advance of modern technologies would bring the new prospective to overcome the bottleneck in the secretion of α-amylase and enhance productivity of heterogeneous proteins in *B. subtilis*.
